# 86. Intravenous anakinra treatment in a rare case of macrophage activation syndrome presenting as fulminant liver failure

**DOI:** 10.1093/rap/rky034.049

**Published:** 2018-09-20

**Authors:** Abeer Ghuman, Kimti Kumar, Animesh Singh

**Affiliations:** Rheumatology, Royal Free Hospital, London, UNITED KINGDOM

**Introduction:** Haemophagocytic lymphohistiocytosis (HLH) is a rare but potentially life-threatening syndrome characterised by abnormal immune activation causing excessive inflammation and tissue destruction. When HLH occurs in the setting of rheumatologic disorders it is termed macrophage activation syndrome (MAS). HLH tends to present with a febrile illness with multiple organ involvement. If left untreated, it has a high mortality due to progressive multi organ failure. A delay in diagnosis due to rarity of the syndrome, variable clinical presentation and the lack of specificity of clinical and laboratory findings are the most common pitfall that leads to progression of disease and poor outcome. Hepatic manifestations of disease are common in MAS, although fulminant liver failure at presentation is rare. Successful liver transplantation in the setting of MAS has only been described once in the literature and is often considered to be contraindicated due to the systemic nature of MAS. The goal of treatment in MAS is to suppress the immune system. The successful use of subcutaneous anakinra as part of treatment for MAS has been reported in limited retrospective studies. We describe a case of macrophage activation syndrome secondary to adult onset Still's disease presenting with fulminant liver failure. The patient went on to have a successful liver transplant. The novel use of high dose intravenous (IV) interleukin 1 receptor anatagonist anakinra to treat MAS is described.

**Case description:** A 21 year old male presented with a three week history of non-specific lethargy, fevers, generalised myalgia and arthralgia. He had noted yellowing of his sclera over the same time course. There was no history of foreign travel or exposure to unwell contacts. He had no cough, dysuria, vomiting or diarrhoea. He had no significant past medical history and was on no regular medication. There was no history of excessive alcohol intake or illicit drug use. Initial examination in the emergency department revealed a fever of 39.2 degrees celsius. He was tachycardic at 112 beats per minute. He was icteric. There was a blanching salmon pink maculopapular rash on his chest, arms and legs that had appeared on the day of presentation. His abdomen was soft but distended and chest examination revealed reduced air entry and sparse crackles. There was no clinical synovitis. Initial blood tests revealed liver failure with bilirubin of 390unit/L, ALT 297unit/L, ALP 179unit/L. There was impaired synthetic liver function with a coagulopathy with international normalized ratio (INR) of 5.9 and low albumin of 21 g/L. Additional blood tests showed a neutrophilia with WCC 25.4 x10*9/L, haemoglobin 138g/L, C-reactive protein 44 and platelets 91 x10*9/L. Renal function was within normal limits. Computed tomography (CT) head was unremarkable. CT chest demonstrated moderate basal atelectasis and pleural effusions. CT abdomen and pelvis demonstrated a grossly abnormal liver with moderate ascites. He deteriorated rapidly over the course of a few hours and was intubated and ventilated and transferred to a tertiary liver centre for consideration of liver transplant. Workup for a cause of acute liver failure revealed a normal paracetamol level, negative hepatitis viral screen, negative EBV IgM and negative autoimmune screen. His ferritin was found to be raised at 16,904 mcg/ml which raised suspicion of macrophage activation syndrome causing fulminant liver failure. His first bone marrow aspirate was negative for macrophage activation in a suboptimal sample. We had a high index of suspicion that this was MAS, as a result treatment for MAS was initiated despite the negative aspirate. He then underwent a second bone marrow biopsy which did show increased macrophage activity with evidence of haemophagocytosis. Triglycerides were unable to be processed as the sample was icteric. D-dimer was raised at 12829ng/ml, lactate dehydrogenase (LDH) high at 401unit/L and fibrinogen was low at 0.5 g/L. Subsequent soluble CD25 was raised at 5879pg/ml. The patient met criteria for diagnosis of MAS on the basis of fever, low fibrinogen, haemophagocytosis in bone marrow, raised ferritin and elevated soluble CD25. MAS secondary to adult onset Still's disease was diagnosed. He fulfilled the Yamaguchi criteria for diagnosis of adult onset Still's disease with fever, arthralgia, rash, leukocytosis and abnormal liver function tests and the exclusion of other rheumatological, infectious or malignant pathology. Initially 100mg subcutaneous anakinra was commenced along with intravenous methylprednisolone at 1g once daily. He was also given intravenous immunoglobulin (IVIg) at 2mg/kg over two days. Despite this, his liver function did not improve and his coagulopathy worsened causing gastrointestinal bleeding. Upper gastrointestinal endoscopy and CT angiogram revealed no focal bleeding point. He underwent an orthotopic liver transplant eight days after commencement of anakinra, steroid and IVIg. The explant showed massive hepatocyte necrosis but no features of MAS. His time zero biopsy of the transplanted liver was within normal limits and his coagulopathy was improved. However within five days of transplant his liver function again began to deteriorate with rising transaminases and a worsening coagulopathy. He underwent a second biopsy of the transplanted liver which showed centrilobular damage with coagulative necrosis and cholestasis. The portal changes were insufficient for a diagnosis of cellular rejection. The worsening liver function and changes seen on biopsy of the transplant were thought to be secondary to ongoing abnormal immune activation from ongoing MAS. The decision was made in conjunction with the transplant team to escalate the anakinra dose over the course of days starting at 200mg to 600mg and given intravenously. The IV methylprednisolone was again pulsed for three days at 1g once daily for three days. He was started on tacrolimus 3mg twice daily post transplant to prevent rejection. The tacrolimus is likely to be having a dual effect of also treating the underlying rheumatic disease as it acts as a potent calcineurin inhibitor similar to ciclosporin which is effective in the treatment of adult onset Stills disease. Anakinra was continued at 600mg IV for a total of four weeks. The methylprednisolone was then continued at lower dose of 100mg once daily. Within four weeks his liver function had returned to normal with ALT 13unit/L, bilirubin 3 unit/L, ALP 10unit/L, albumin 32g/L and INR 1.0. His ferritin reduced to 463 mcg/ml over the course of six weeks. He required a tracheostomy which was then successfully weaned and decannulated. His post intensive therapy unit (ITU) stay was complicated by pneumonia, deep venous thrombosis and pulmonary embolus requiring anticoagulation with a novel oral anticoagulant. He required haemofiltration for elevated ammonia pre and post liver transplant. The anakinra was reduced to 200mg subcutaneous once the ferritin was below 1000 mcg/ml and he was put onto a weaning dose of oral prednisolone starting at a dose of 40mg. His liver function remains normal. He is now undergoing inpatient rehabilitation for ITU critical illness neuropathy. He remains on 12.5mg prednisolone and 200mg subcutaneous anakinra. His inflammatory markers are normal and his ferritin remains below 500 mcg/ml.

**Discussion:** We have described a case of macrophage activation syndrome presenting as acute liver failure in a previously well young adult patient. Escalation to treatment with intravenous interleukin 1 receptor antagonist anakinra, in addition to intravenous immunoglobulin, corticosteroids, tacrolimus and liver transplantation led to a positive outcome. Rapid access to intravenous anakinra at our centre facilitated a novel therapeutic and multidisciplinary approach to treatment of this condition. This case emphasises the importance of early diagnosis of the condition and maintaining a high index of suspicion, due to the rapid progression of unidentified and untreated disease. Importantly, in this case, treatment with anakinra was then initiated early despite the absence of diagnostic certainty, once sepsis had been excluded. Treatment with anakinra was then escalated to intravenous formulation on the presumption of better absorption in the setting of liver transplant failure. There are several potential treatment regimens that have been suggested for HLH, all of which are based on observational studies and thus evidence for these is limited. The efficacy of supportive care in this condition is poor. Anakinra has been previously described in treatment, although most commonly in a subcutaneous formulation, rather than as intravenous administration as provided in this case.In situations such as this where a diagnosis of underlying rheumatological disease is made concurrently with the diagnosis of HLH, or MAS, diagnosis can be particularly difficult. Several diagnostic features of this case used to make a diagnosis of HLH are also clinical findings of adult onset Stills disease and careful history of preceding illness and examination were integral to establishing both diagnoses. There were several expert teams involved in the care of this patient, including rheumatology, haematology, hepatology, hepatobiliary transplant surgery and ITU. This coordinated multidisciplinary care was integral to the positive outcome of this complex case.

**Key Learning Points:** Consider HLH/MAS as a cause of acute fulminant liver failure. Use of escalating IV doses of anakinra for HLH/MAS. Bone marrow aspirate can be negative in HLH so a negative result doesn’t exclude HLH. Repeat aspirates may be required. Early diagnosis of haemophagocytic lymphohistiocytosis and a high index of suspicion required. Focused history and examination looking for underlying rheumatological disease, to suggest macrophage activation syndrome. Treatment in centre with multidisciplinary care including advanced biologic treatment. Early initiation of treatment based on a high likelihood of HLH versus diagnostic certainty. There is a high mortality rate with this condition if untreated. Efficacy of interleukin 1 receptor antagonist, anakinra, of intravenous formulation, in combination with IVIG and glucocorticoids Further research and clinical trials are required.

**Disclosure: A. Ghuman:** None. **K. Kumar:** None. **A. Singh:** None.

